# “*Libre de Gluten*” or “Gluten-Free”? The Influence of Loanwords on Conceptual Representation

**DOI:** 10.3390/foods15152686

**Published:** 2026-07-30

**Authors:** Claudia Ariadna Acero-Ortega, Aurora Pintor-Jardines, Oxana Lazo, Iván Méndez, José M. Remes-Troche, Sergio Erick García-Barrón

**Affiliations:** 1 ESDAI, Universidad Panamericana, Augusto Rodin 498, Ciudad de México 03920, Mexico; cacero@up.edu.mx; 2 Departamento de Biotecnologia, Universidad Autonoma Metropolitana Iztapalapa, Av. San Rafael Atlixco 186, Leyes de Reforma 1ra Secc, Iztapalapa, Ciudad de México 09340, Mexico; ma.pintorj@izt.uam.mx; 3 Centro de Investigación en Biotecnología Aplicada, Instituto Politécnico Nacional, Carretera Estatal Santa Inés Texcuexcomac Km 1.5, Tepetitla 90700, Tlaxcala, Mexico; olazoz@ipn.mx; 4 MBSense, José María Vertiz 847, B403, Ciudad de México 03023, Mexico; ivan.mendez@mbsense.com; 5 Laboratorio de Fisiología Digestiva y Motilidad Gastrointestinal, Instituto de Investigaciones Médico Biológicas, Universidad Veracruzana, Calle Agustín de Iturbide, Salvador Díaz Mirón, Veracruz 91700, Mexico; jose.remes.troche@gmail.com

**Keywords:** gluten-free foods, social representations, consumer behavior, Anglicism, celiac disease

## Abstract

The expansion of the gluten-free food market is driven by two distinct factors: medical prescriptions for people with celiac disease, and lifestyle adoption among non-celiac consumers. This study examines how the use of the English term “gluten-free” may influence the cognitive conceptualization of the product compared to the term “*libre de gluten*” among two groups. Methodologically, the cognitive structure of 240 Mexican consumers (120 with celiac disease and 120 without) was evaluated using social representation theory and the word association technique. Findings reveal that the language used on labels significantly influences the semantic categories evoked. The non-celiac group exhibits an intellectualized and stable representation for both variants, prioritizing health and composition. In contrast, the celiac group experiences a dichotomy: the Spanish version triggers a restrictive reality linked to the consumption experience and economic factors, while Anglicism may shift the perception towards health and aspirational dimensions. It is concluded that the linguistic framework of the labeling operates as a distinct cultural vehicle; Anglicism denotes status for the non-celiac consumer but may evoke an aspirational mechanism that mitigates disease barriers in the celiac patient.

## 1. Introduction

In recent years, the number of consumers following a gluten-free diet has increased, partly due to the increased prevalence and awareness of gluten-related disorders (GRDs), in particular celiac disease (CD), which has become a global public health issue [1,2,3]. In addition, there has also been an increase in the number of consumers following a gluten-free diet due to a health halo effect [1,4,5]. According to Hassan et al. [2], the global demand for gluten-free foods (GFFs) has increased 16% from 2018 to 2022, making it one of the top 10 food trends. This increase has transformed the GFF market from a specialty niche to a global one in the food sector [3]. Nevertheless, this expansion comes with a significant gap between epidemiology and consumption. While gluten-related disorders affect only a minority of the global population [6], up to 25% of these consumers purchase these products without a medical diagnosis [4]. This phenomenon is driven by self-diagnosis and extensive media coverage [7], which has contributed to the spread of the unfounded belief that GFFs are healthier or more suitable for weight management [3].

In Mexico, where celiac disease was considered rare less than a decade ago, the proliferation of these types of foods (which are mainly imported) has forced authorities to update regulations, standardize labeling, and protect an increasing population influenced by this major trend [8]. Consequently, research and development into GFFs has increased, making them resemble the original products containing gluten in order to meet consumer demands [1,9].

As part of the development process, it is important to address the subjective perspective of consumers, as this may provide further insight into the factors that influence GFF consumption [1,3]. In this sense, the analysis of the representation of the term “gluten-free” can be useful for identifying consumers’ feelings, beliefs, attitudes, motivations, and opinions about this term [1].

Considering that GFFs are consumed by both celiac and non-celiac individuals, it is important to know how they are perceived and to identify the barriers and realities that separate these two consumer groups. For patients with celiac disease, a gluten-free diet presents a daily medical challenge marked by limited product availability, high costs, and poor sensory quality [6,10]. Furthermore, they face a market where misleading labeling and cross-contamination pose a real risk to their health [8].

According to do Nascimento et al. [10], celiac consumers perceive GFFs as high-energy, high-fat foods that contain numerous additives and a long list of ingredients. In contrast, non-celiac consumers adopt these foods as a lifestyle choice driven by the health halo effect, perceiving them as healthier, lower in calories, and less processed [4]. This belief often leads them to overlook the fact that industrial gluten-free substitutes may compensate for the lack of gluten with higher amounts of sugar and fat, resulting in a product with an inferior nutritional profile [4].

Correspondingly, it is crucial to understand how product information is conveyed, particularly through food claims, which can influence how consumers perceive and behave towards products. In a Mexican context, finding products labeled as “*libre de gluten*” or “gluten-free” is quite common. This suggests a penetration and assimilation of foreign expressions from other languages whose influence and significance at the sociocultural level may differ among different social groups [11].

This type of linguistic exchange can lead to variations in both the distribution of the target language’s vocabulary and its lexical inventory. Among the modifications to the inventory that affect both content and expression are so-called linguistic loans [12]. Linguistic loans are defined as words, expressions, or morphemes that are adapted from another language and incorporated into the target language, potentially altering their form or meaning [13]. This is a natural result of language contact, a universal phenomenon observed in all languages [14]. According to Silva-Corvalán [15], linguistic behavior differs between men and women and is influenced by age and social groups.

This indicates that social factors regulate linguistic behavior, as well as the linguistic attitudes within a speech community. Furthermore, in the context of food marketing, foreign languages are often used for symbolic purposes rather than literal comprehension, acting as conveyors of cultural values. The use of English vocabulary, such as the term “gluten-free”, is frequently and strategically employed to transfer cultural associations to the product, thus creating an international, modern, and cosmopolitan image [16].

Therefore, it is necessary to analyze the modification that linguistic loans can have on the representation of a concept such as the term “gluten-free”. In the Mexican market, the English “gluten-free” may be related to aspirational symbols, linking products to prestige and modern lifestyles. This Anglicization may trigger divergent interpretations: medical safety for celiacs versus social identity for non-celiacs. Thus, it is crucial to determine if this linguistic loan and its Spanish translation share the same representational core or evoke different realities. In this vein, projective techniques can be useful for understanding consumer perception of a stimulus of interest [17], such as the word association test (WAT). The WAT allows for the discovery of latent or subconscious aspects of behavior related to a stimulus [18], while words are generated spontaneously from a verbal or visual stimulus [19]. In the context of GFFs, Capriles et al. [1] point out that the use of the WAT to obtain consumer opinions has been limited as previous research has focused on different objectives, such as expectations [20], consumer perceptions of GFFs’ claims [21], and perceptions of GFF availability on the market [1,3]. Therefore, it is important to understand how celiac and non-celiac consumers conceptualize the term “gluten-free”.

As a result, this article draws on the theory of social representations (TSR). TSR aims to understand the knowledge and attitudes of a social group towards different aspects, cultural areas, or concepts that lack clarity [19], focusing on the development of the representation of a social object within a social group [22,23]. This theory has been used to assess consumer behavior in various contexts [24,25,26,27,28,29,30], such as analyzing and defining the structural approach of social representations [26,31]. This method holds that a social representation is organized around a central core, which is decisive in the meaning and organization of the representation [32]. The central core has both a meaning-generating function and a meaning-organizing function; it provides purpose to the entire representation and determines the nature of the relationships between its elements. Finally, it defines the object of representation and the social practices related to it [33,34,35]. Therefore, the objective of this study was to analyze the social representations of the term “gluten-free” in both English and Spanish among celiac and non-celiac consumers.

## 2. Materials and Methods

### 2.1. Questionnaire Design

The study was conducted following the Code of Ethics established under the Declaration of Helsinki for tests involving humans. The questionnaire was approved by the ethical committee of the Instituto de Investigaciones Medico-Biologicas, with registration number IIMB-UV-2025-14. An online questionnaire was developed and previously validated with a pilot test. The questionnaire consisted of three sections and was elaborated using FIZZ software (version 2.82 a 03 Biosystemes 1994-2025). First, a brief description of the study was given. Participants gave their informed consent by clicking on the start box to begin the survey. The first section consisted of the word association test (WAT) to collect the concepts that the participants associated with the terms “*libre de gluten*” and “gluten-free”. The second section was oriented to collect the sociodemographic information of the participants, such as level of education, age, experience with gluten-free products, and familiarity with the gluten-free concept.

### 2.2. Participants

The study was conducted between August and December 2024. The sampling focused on two groups of participants: 120 non-celiac consumers and 120 celiac consumers. In the case of non-celiac consumers, surveys were conducted by inviting participants via social media to participate in the study, with the inclusion criterion being the consumption of gluten-free products. Online administration of tests such as the WAT is reliable and stable, ensuring that results are comparable to responses obtained from in-person tests [36]. In the case of celiac consumers, the surveys were administered through celiac associations in Mexico and patients at the Laboratorio de Fisiologia Digestiva y Motilidad Gastrointestinal at the Universidad Veracruzana, Veracruz, Mexico.

### 2.3. Word Association Test

Participants who agreed to take part in the study were informed that there were no right or wrong answers. The terms “*libre de gluten*” and “gluten-free” were used as textual stimuli. To elicit associations, both participants with and without celiac disease were asked the following question: Can you name four words that come to mind when you read the expression “gluten-free”? The same question was asked separately for both terms in English (“gluten-free”) and in Spanish (*libre de gluten*). To avoid potential bias, the order in which the two terms were presented was randomized. Afterwards, they were asked to rank the four words evoked from most important (1) to least important (4), taking into account each one of the stimuli evaluated. Participants were then asked to rate the attitude level of each word mentioned towards the concept being evaluated, using a seven-point scale ranging from −3 (completely negative) to +3 (completely positive) [24,25]. Finally, they were asked to indicate how familiar they felt with each of the concepts evaluated. To achieve this, a seven-point scale ranging from 1 = not very familiar to 7 = very familiar was used.

### 2.4. Data Analysis

#### 2.4.1. Familiarity Analysis

To analyze the role of consumers’ self-perceived familiarity with gluten-free foods, a one-way ANOVA was performed. Health status (celiac and non-celiac) was considered a fixed factor, while participants were considered a random factor to evaluate significant differences (*p* < 0.05).

#### 2.4.2. Pretreatment of Evoked Terms

Before performing the analyses, spelling and typographical errors were reviewed and corrected for both consumer groups. In this context, two data matrices were constructed, one for non-celiac consumers and another for celiac consumers. Each database included the list of words obtained for each stimulus, participant, age, gender, experience with the product, and time of day when the food was consumed. Next, a lemmatization process was carried out to correct and normalize the words, following Bécue-Bertaut, Álvarez-Esteban, and Pagès [37]. The words were then grouped into synonyms using a thesaurus. The most frequently used words were used to group all their synonyms. For ambiguous words that were not easy to classify, the researchers decided by consensus whether they could be regrouped or left as uncategorized words. Subsequently, the words were translated from Spanish to English using a double translation. The English translation was then back-translated into Spanish. If the translation was correct, it was kept, but if the translation changed, the reviewers made sure that it had the same meaning in Spanish.

#### 2.4.3. Semantic Categorization

The total number of words was subjected to a grouping process based on the personal interpretation of the researchers who participated in the study. The process was carried out using triangulation [38], in which three researchers performed the analysis independently. The researchers then shared and agreed on the final semantic categories. This grouping process was carried out in accordance with the methodological guidelines established in various studies that have used the word association test [19,24,25,28,30]. For subsequent analyses, only categories with frequencies greater than 2% were considered, since an inferior value could be considered a low-mention threshold [39]. To verify the presence of statistically significant differences between the frequencies of the semantic categories for each of the terms evaluated by both consumer groups, a K-proportions test was conducted using the Marascuilo procedure (*p* < 0.05). This nonparametric statistical test is used to determine whether the observed response proportions differ significantly among three or more groups—in our case, semantic categories [40].

#### 2.4.4. Analysis of the Representation

In the structural analysis, words that were mentioned by at least 10 participants were taken into account [41]. For each concept within each group of participants, the total frequency and average importance of the words retained in each condition were calculated. The cutoff point of importance was calculated by averaging the ratings of the evoked words. For the frequency cutoff point, the maximum difference between two successive frequencies was taken [24,28,30]. Subsequently, the social representation map was constructed. The map consisted of four zones (2 × 2) which were delimited by the cutoff points. The first zone contained the most important and most frequently cited terms, which stands for the core of the representation. The second zone is the first periphery and contains words with less importance but more citations. The third zone, or contrast zone, contains terms of great importance but with low citation frequency. Finally, the fourth zone, or second periphery, contains the least important and least cited terms.

#### 2.4.5. Polarity Index

To define the positive or negative valence of the representation, a polarity index (P-index) [42] was estimated. The index was calculated as the ratio between the difference in the number of positive and negative words and the total number of words evoked. The value of the index ranges from −1 to +1. Values between −1 and −0.4 indicate that the word has a negative connotation; values between −0.4 and +0.4 indicate that the word has a neutral connotation; and values between +0.4 and +1 indicate a positive connotation [25]. To determine attitudes towards the social representation of each term, the P-index was calculated [25,42]:

$$P=\frac{Number\;of\;positive\;words-Number\;of\;negative\;words}{Number\;of\;total\;evoked\;words}.$$

#### 2.4.6. Health Condition and Correlation with Terms

To verify the relationship between the evoked words and health conditions (celiac or non-celiac), a correspondence analysis (CA) was performed. For this purpose, a four-by-twenty-nine contingency table was constructed. Rows represented the participants’ health condition, and the 29 words that made up the conceptual structure were considered columns. Pearson’s chi-square test was used to verify the relationship between health condition and the words considered. Afterwards, a hierarchical cluster analysis (HCA) was performed using Ward’s agglomerative method using the Euclidean distance of the columns’ principal coordinates obtained from the CA. This combined approach enables the identification of similarities and association patterns among the four experimental conditions, rather than individual participants. The analysis was performed using XLSTAT 2020 ver. 5.1 software (Addinsoft, New York, NY, USA).

## 3. Results

### 3.1. Participants

Table 1 presents the sociodemographic characteristics of the participants, as well as their consumption patterns and levels of familiarity with gluten-free products. In both groups, women outnumbered men, accounting for 81.7% of the celiac group and 63.3% of the non-celiac group. Regarding educational level, 87.5% of celiac consumers had a college education, compared with 62.5% of non-celiacs with the same level of education. The age distribution shows that the celiacs in this sample were primarily aged between 25 and 54 years old, while non-celiacs were concentrated in the 18 to 34-year age range. Regarding experience with product consumption, the 1-to-7-year range predominates among celiacs, with 11.7% declaring more than 10 years of continuous consumption. In contrast, the largest percentage of non-celiacs had between 1 and 3 years of experience or less than 1 year of experience with GFFs. Finally, regarding their level of familiarity with gluten-free products, celiac consumers showed a higher degree compared to non-celiacs, with average scores of 5.7 and 4.2, respectively, with a statistically significant difference (*p* < 0.05). This difference in familiarity reflects the contrasting nature of consumption between the two groups: a mandatory long-term clinical restriction in the celiac group, versus a recent adoption driven by lifestyle trends in the non-celiac group. For this reason, familiarity was used exclusively as a descriptive indicator of potential exposure to the products and not as a covariate for statistical adjustment.

### 3.2. Semantic Categories

**Figure 1:**
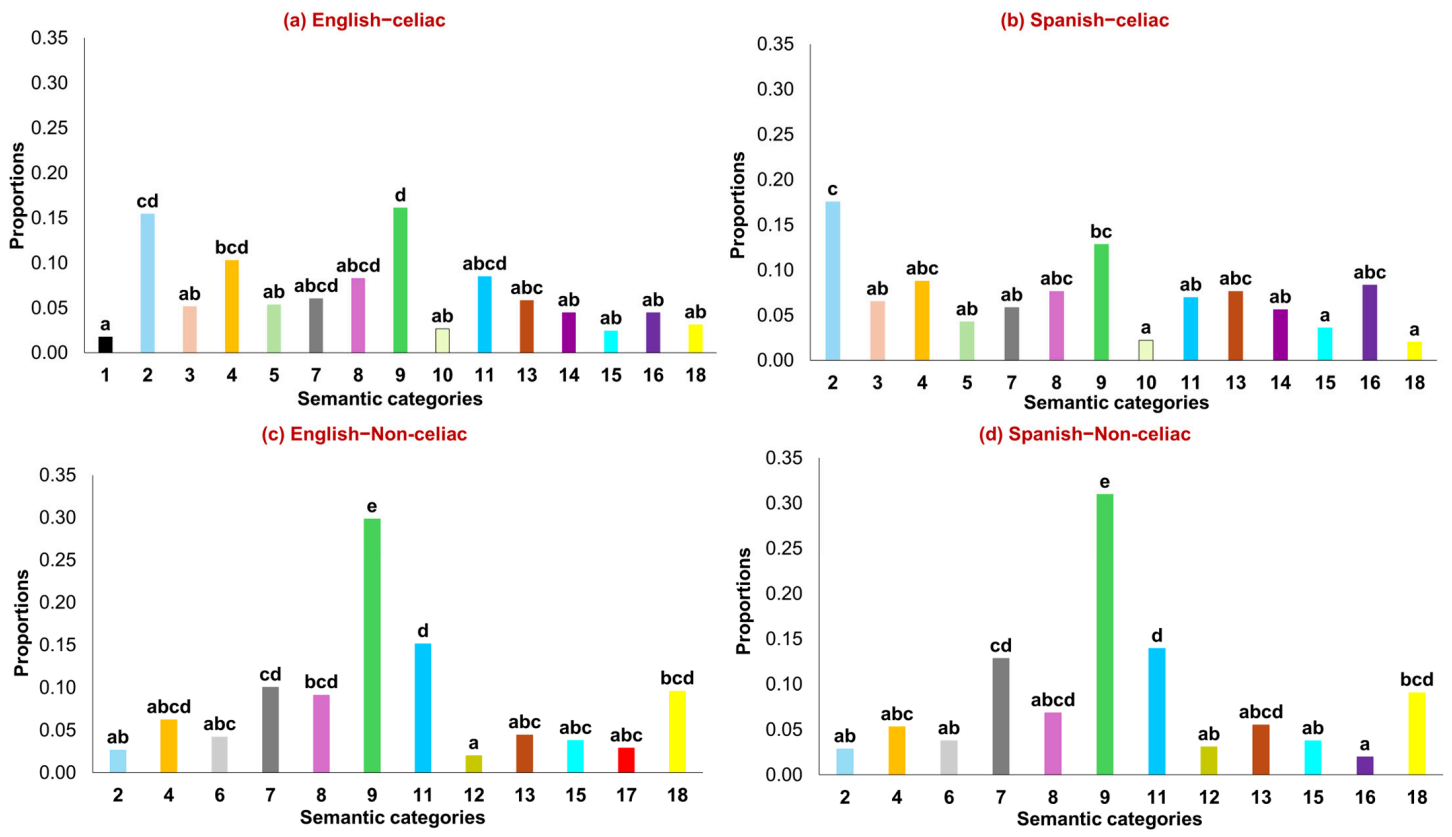
Semantic categories of the “*libre de gluten*” and “gluten-free” terms by the celiac and non-celiac experimental groups. Nomenclature: 1 = Aspirational aspects; 2 = Consumption experience; 3 = Doubt/unknown; 4 = Economic elements; 5 = Extrinsic attributes; 6 = Food composition; 7 = Food products; 8 = Free-from; 9 = Health aspects; 10 = Hedonic features; 11 = Ingredient composition; 12 = Innovation 13 = Intrinsic attributes; 14 = Legislation; 15 = Manufacturing processes; 16 = Market availability; 17 = Marketing; 18 = Nutritional aspects. Different letters above each bar indicate significant differences between the semantic categories, as determined by the chi-square test (*p* < 0.05) or K-proportions test using the Marascuilo procedure.

Following analysis of the corpus, its coding, and the triangulation process, a total of 480 evoked words were obtained for each consumer group (people with celiac disease and those without), taking into account the stimuli in English and Spanish. The words were then organized into semantic categories, resulting in an initial total of 22 categories. However, for subsequent analyses, only those categories that exceeded a 2% frequency of mention were retained. This resulted in a final set of 18 semantic categories, thereby ensuring the relevance of the analyzed associations. Figure 1 shows the semantic categories used to group the terms “gluten-free” and “*libre de gluten*” among the celiac and non-celiac experimental conditions. Among the most frequently cited categories were consumption experience, health aspects, economic elements, ingredient composition, and food products.

The results show that nine semantic categories are common to all four evaluation conditions, four categories are specific to consumers with celiac disease, two are specific to participants without celiac disease, and there was only one category specific to the “English—celiac” condition (aspirational aspects) and the “English—non-celiac” condition (marketing), which reflects that each condition has its own distinct profile in terms of semantic categories. According to the K-proportions analysis, the language of the stimulus significantly influences the semantic categories evoked by consumers. Among celiac consumers, when evaluating the term “*libre de gluten*”, the most frequently evoked semantic category was consumption experience (17.6%). However, when the term “gluten-free” was presented, the priority shifted, with health aspects taking first place at 16.1%. This aligns with the findings for non-celiac consumers, for whom the health aspects category had the highest frequency, at 31% and 29.8% for the Spanish and English terms, respectively.

A notable finding is that, among celiac consumers, the English format (“gluten-free”) elicited responses aligned to the Aspirational category, which accounted for 2% of responses. Notably, this category did not emerge when the stimulus was presented in Spanish, nor did it appear among non-celiac consumers. Furthermore, for celiac consumers, the Economic category maintained a strong presence in both languages, though its frequency increased with the English term (10.3%) compared to the Spanish one (8.8%). In the case of non-celiac consumers, the categories Ingredient Composition and Food Products also stood out for the term “*libre de gluten*” (11.39% and 12.8%, respectively) compared to 15.1% and 10% for “gluten-free”. For both Spanish and English presentation formats, the categories with the lowest recall scores among celiac consumers were Nutritional aspects (2.0–3.1%), Hedonic aspects (2.3–2.7%), Manufacturing Processes (2.5–3.6%), and Legislation (4.5–5.6%). Meanwhile, Market Availability showed a consistently low and identical evocation rate in both languages (4.5%). Regarding non-celiac consumers, the categories with the lowest evocation rates were Market Availability (2%) and Consumption Experience (2.9%). Categories that proved to be more relevant for non-celiac consumers were Innovation (3.1%) and Food Composition, both at 3.8%. On the contrary, the Hedonic category was only important for the celiac consumers.

### 3.3. Structural Approach

#### 3.3.1. Social Representation of Celiac and Non-Celiac Consumers

**Figure 2:**
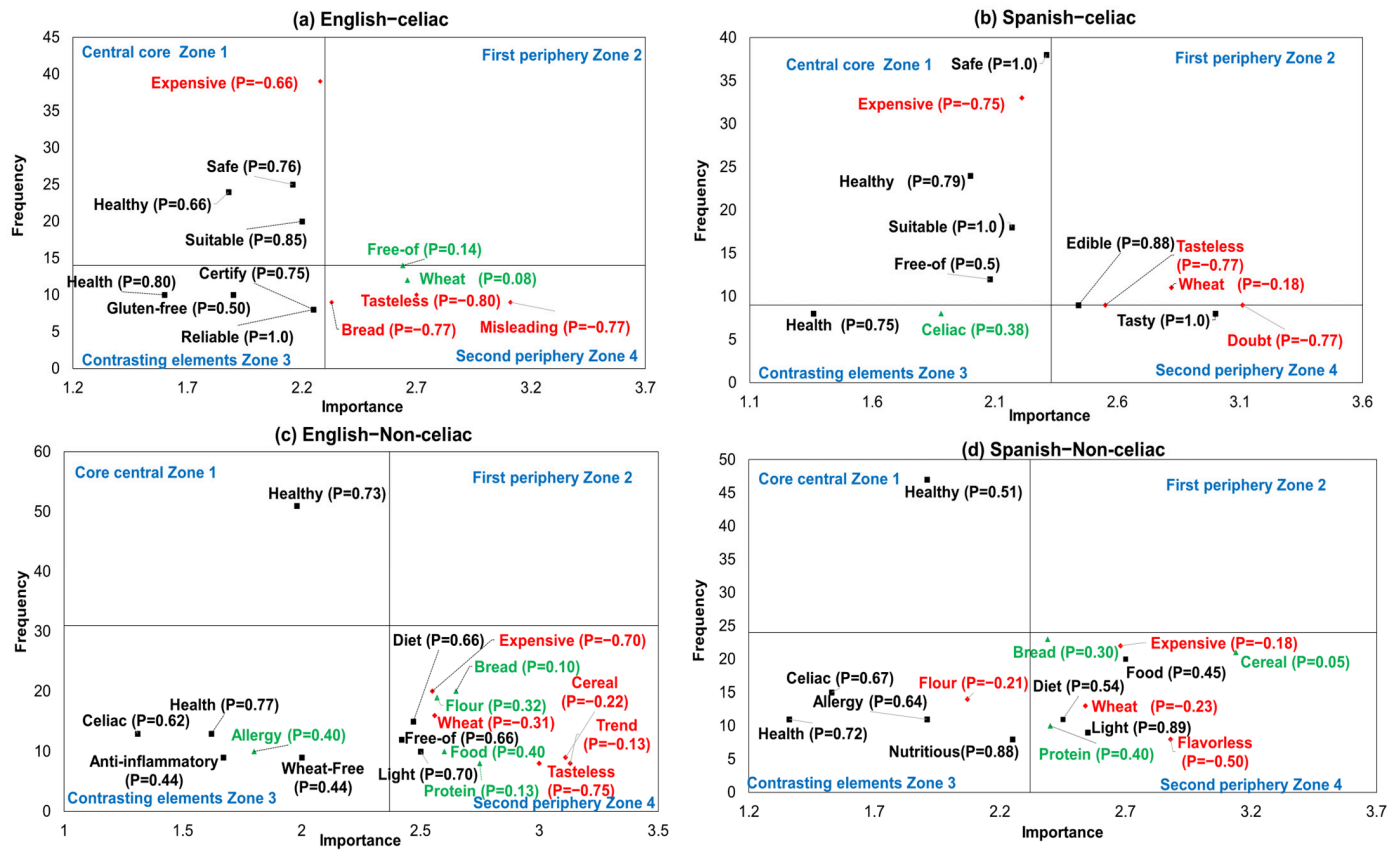
Prototypical analysis of the representation of the terms “gluten-free” and “*libre de gluten*” in the celiac population (**a**,**b**) and the non-celiac population (**c**,**d**). Colors indicate the polarity of the words: red = negative polarity, green = neutral polarity, and black = positive polarity.

Figure 2 shows the results of the prototypical mapping and the P-index (P) of the evocations, broken down by group and linguistic stimulus. The cutoff frequency indicates the degree of agreement among celiac and non-celiac participants; lower values indicate lower agreement between the groups. The four experimental conditions exhibited different cutoff frequencies; however, the highest frequencies were observed in the non-celiac group: 32 for English and 24 for Spanish, compared to 14 for English and 9 for Spanish in the celiac group. This indicates greater agreement within the non-celiac group. Another parameter shown in Figure 2 is the P-index (P), which indicates the emotion perceived by the participants; negative values were associated with a negative attitude, and positive and high values with a positive attitude.

The four maps showed distinct zones depending on the consumers’ medical condition. In the case of celiac consumers, their central core is highly different and diverse (Figure 2a,b). Words that make up the structural representation of celiac consumers include Healthy (P = 0.66 in English and P = 0.79 in Spanish), Safe (P = 0.76 in English and P = 1.0 in Spanish), Suitable (P = 0.85 in English and P = 1.0 in Spanish), and Free-of (P = 0.50 in Spanish only). A term that stands out in the representation for celiacs due to its negative polarity is Expensive (P = −0.66 in English and P = −0.75 in Spanish). In the case of non-celiacs (Figure 2c,d), their central core is based solely on the term Healthy, which exhibits high frequency and positive polarity in both English (P = 0.73) and Spanish (P = 0.51).

Regarding the first periphery (Zone 2), it is empty across almost all four methodological conditions. This indicates that none of the words evoked by consumers met the criteria of high frequency and low importance, suggesting that the central core of the two groups lacks strong elements to protect them against new information. The only visible evocation in Zone 2 occurred in the Spanish stimulus condition for the celiac group, which was Wheat with a P = −0.18. An important observation in the maps, particularly for celiacs, is that there are some terms straddling the line between Zone 2 and Zone 4. In the case of the English stimulus for celiacs, it is Free-of with P = 0.14, while for the Spanish stimulus, there are three terms: Edible (P = 0.88), Tasteless (P = −0.77), and Doubt (P = −0.77). These elements are known as transitional or borderline terms, which will be discussed later.

Based on the contrasting elements in Zone 3, celiac consumers used fewer terms than non-celiac consumers. Terms evoked by celiac consumers under the English stimulus condition with a positive valence were Health (P = 0.80), Certify (P = 0.75), Gluten-free (P = 0.50), and Reliable (P = 1.0). Conversely, celiac consumers under the Spanish stimulus condition used only the terms Health (P = 0.75) and Celiac (P = 0.38). In the case of non-celiacs with the English variant, we can observe terms with high polarity, such as Health (P = 0.77), Celiac (P = 0.62), Anti-inflammatory (P = 0.44), and Wheat-Free (P = 0.44). Allergy (P = 0.4) was mentioned with lower polarity. Finally, non-celiac consumers under the Spanish condition indicated the following terms with positive polarity: Celiac (P = 0.67), Allergy (P = 0.64), Health (P = 0.72), and Nutritious (P = 0.88). Only one term with negative polarity was mentioned, which was Flour (P = −0.21). Finally, for the celiac group under the English stimulus condition, the following terms with negative polarity were observed in the second periphery (Zone 4): Tasteless (P = −0.80), Misleading (P = −0.77), and Bread (P = −0.77). The term Wheat (P = 0.08) was also used with low but positive polarity. For celiacs under the Spanish stimulus condition, Tasty (P = 1.0) and Doubt (P = −0.77) were used.

According to the results for non-celiac consumers under the English stimulus condition, the following terms with positive polarity were observed in Zone 4: Diet (P = 0.66), Free-of (P = 0.66), and Light (P = 0.70). Terms with positive but low polarity included Bread (P = 0.10), Flour (P = 0.32), Food (P = 0.40), and Protein (P = 0.13). Conversely, terms perceived as having a negative connotation were Expensive (P = −0.7), Wheat (P = −0.31), Cereal (P = −0.22), Trend (P = −0.13), and Tasteless (P = −0.75). Finally, for Zone 4 among non-celiac consumers under the Spanish stimulus condition, terms with positive polarity were used, such as Food (P = 0.45), Diet (P = 0.54), Light (P = 0.89), Bread (P = 0.30), Protein (P = 0.40), and Cereal (P = 0.05), although the latter had a low positive polarity. On the other hand, terms with negative connotations included Expensive (P = −0.18), Wheat (P = −0.23), and Flavorless (P = −0.50).

#### 3.3.2. Correspondence Analysis

**Figure 3:**
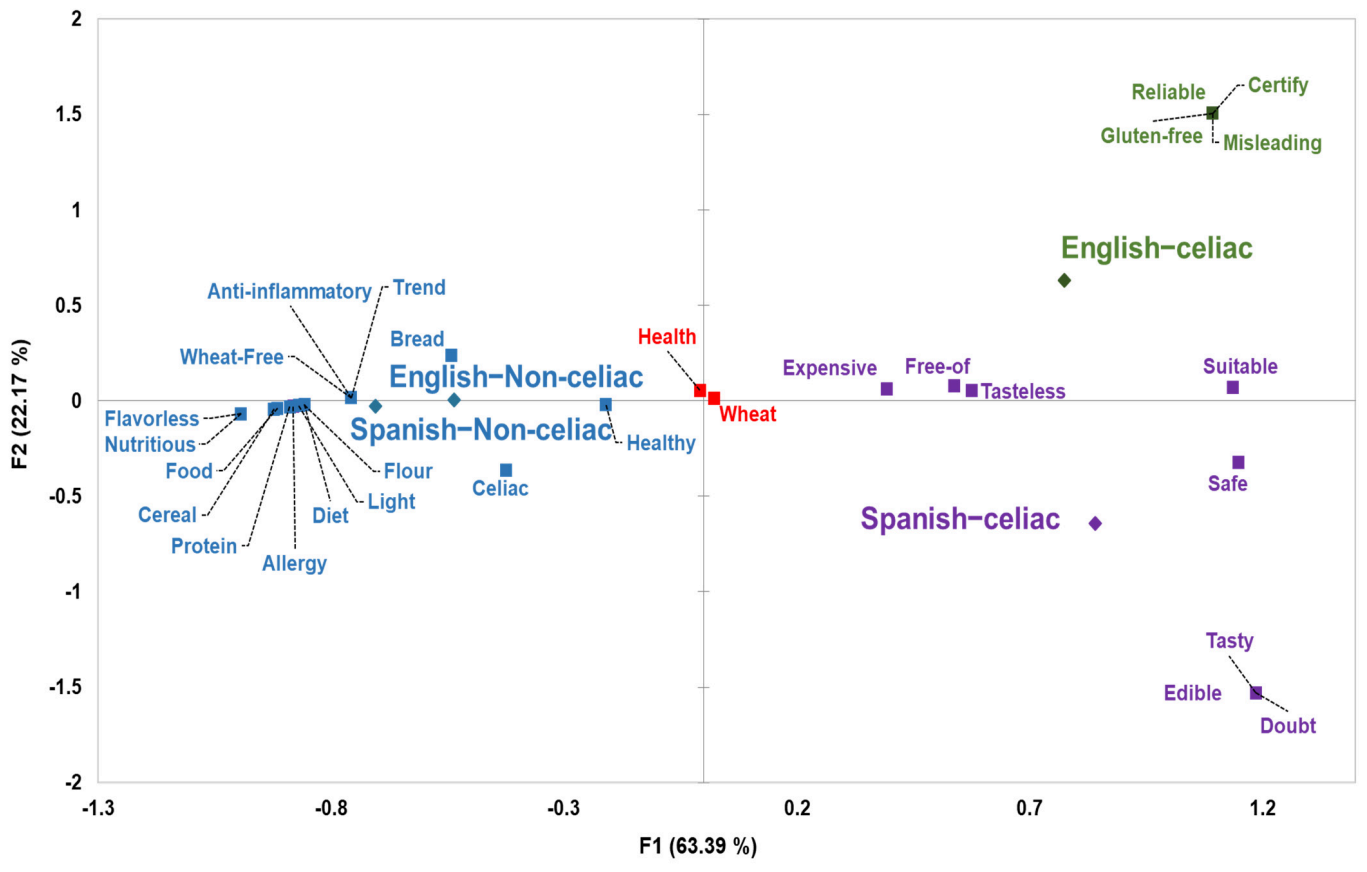
Correspondence analysis of words and conditions. Squares correspond to words and diamonds to conditions. The hierarchical clustering of the CA coordinates proves that variables (words) can be related with the experimental condition (celiacs and non-celiacs). Squares in blue represent words from the non-celiac consumers in both Spanish and English terms, green squares correspond to words from celiac consumers associated with the English term; violet squares represent words associated with celiac consumers using the Spanish term; and red squares indicate words shared across both consumer groups and languages.

This analysis shows the word associations for each consumer group studied. The first two dimensions (F1 and F2) accounted for 85.56% of the total variance, with 63.39% attributed to F1 and 22.17% to F2. This indicates that the graph accurately reflects most of the variance (Figure 3). The primary axis F1 clearly separates the medical conditions of the participants. All consumers with celiac disease, regardless of whether the stimulus was in English or Spanish, are located on the right part of the graph. These consumers are almost completely separated from those without celiac disease, who are situated on the left side of the biplot. Furthermore, for consumers with celiac disease, the F2 axis separates their associations based on the language of the stimulus. This shows that associations for celiacs in English terms are placed in the top right side, while associations for celiacs presented with Spanish terms are in the middle and bottom of the right side of the biplot. When the stimulus was presented in English, words from consumers with celiac disease are located in the upper-right quadrant, with perceptions grouped around concepts related with the food industry. They used words such as reliable, certify, gluten-free, and misleading.

In contrast, in the lower-left quadrant, when the stimulus was in Spanish (their native language) the mental schema of celiacs shifted towards the bottom of the graph. This revealed a focus on the daily reality of their condition and the barriers they face. The words evoked here were associated with specific characteristics of the food and their medical condition, using terms such as expensive, free-of, bland, suitable, safe, doubt, edible, and tasty. As for non-celiac consumers, under both stimulus conditions, they were grouped in the central area of the graph. This indicates that, regardless of language, consumers share a common view of gluten, immediately associating it with Bread, Wheat, and Health. This centrality suggests that, for non-celiacs, gluten is not considered a problematic element, but rather an everyday component related to basic nutrition and well-being.

On the other hand, there is clear polarization in the celiacs group, which shifts notably toward the right quadrant of the F1 axis (accounting for 63.39% of the variance). While non-celiacs occupy a neutral zone, celiacs (in both English and Spanish) aligned their perceptions with critical safety attributes. This contrast is significant for the healthy consumer (non-celiac), as they view the product as a lifestyle choice. For the celiac, however, it represents a need for risk management, characterized by doubt and the search for technical validation. It is interesting to note that the concepts of health and wheat are located at the center of the graph for all four conditions.

## 4. Discussion

This study examines the effect of the use of loanwords on the representation of the term “gluten-free”. For this purpose, a survey was conducted among consumers of gluten-free products with two different health conditions: consumers with celiac disease and consumers without celiac disease (non-celiac).

### 4.1. Participants

Differences in gender and education status between participants with celiac disease versus non-celiacs appear to be unbalanced. Nevertheless, it is important to consider that the identified celiac population is still rather low around the world and especially in countries like Mexico, making it difficult to gain a balanced sample for both groups. This fact could be addressed further.

The results presented in Table 1 show that the celiac group has a significantly higher level of familiarity (5.7) with the evaluated products compared to the non-celiac group (4.2), suggesting that perceptions of gluten-free products differ between the two groups. This is consistent with the findings of García-Barrón et al. [28], who noted that continuous exposure to a stimulus and the level of familiarity with it determines the consensus and structure of its social representation. These differences in familiarity are likely related to the duration of adherence to the diet: in our study, consumers with celiac disease have followed a strict diet for several years (between 4 and 10 years), which has shaped their perspective based on the difficulty of dealing with these products over time. This prolonged experience could explain the “aspirational aspects” identified in this study.

Among non-celiac consumers, 58.9% had recently been consuming gluten-free foods, suggesting that this trend may be derived from fashion or novelty. This aligns with the sociological perspective described by Lo Monaco and Bonetto [23], in which food choices function as positional goods for status and shape the social identity of the individual. Similarly, these consumers react positively to the Anglicism “gluten-free”, attributing a role as a conveyor of cultural values to the foreign language that gives food an international, modern, and cosmopolitan image [16].

Although English proficiency was not directly measured among participants, differences between “gluten-free” and “*libre de gluten*” were based on consumers’ reactions towards the Anglicism or the native phrase, regardless of their language knowledge. Nevertheless, further studies could benefit from having an additional measurement of this variable for possible representation leverage.

### 4.2. Structural Approach

#### 4.2.1. Semantic Categories Analysis

After reviewing the differences in the semantic categories mentioned by both consumer groups, it is clear that these are not merely lexical variations, but rather two distinct ways of constructing a reality around food. According to García-Barrón et al. [28], the most frequently cited categories reflect their importance in consumers’ minds and are responsible for defining the evaluated object. Thus, among non-celiac consumers, the dominant categories were Health, Ingredient composition, Food products, and Nutritional aspects. This indicates that the representation of gluten-free food for this group is more intellectual and symbolic, based on label claims and the cultural value associated with the term, thereby distancing it from the food itself [16]. In this group, the product is perceived both as food and as a symbol associated with health, well-being, and a certain lifestyle.

In contrast, the perception among consumers with celiac disease is shaped by their restrictive reality, prioritizing categories such as Consumption experience and Economic aspects. This suggests that their shared understanding is based on overcoming barriers such as high costs and poor sensory qualities [6,10]. Unlike non-celiac consumers, the nutritional composition of food loses importance compared to the need to find safe, accessible, and sensory-acceptable products. This difference shows that the meaning attributed to the gluten-free food depends on the product itself, its function, and the emotion that each group associates with it. Based on the proportion analysis conducted, it was also found that the language of the stimulus alters the priority of categories among celiac consumers. When the term “*libre de gluten*” was used, the most frequently evoked semantic category was Consumption experience, which aligns with studies by do Nascimento et al. [10] and Alencar et al. [3]. Despite market growth, celiac consumers still express dissatisfaction with the texture and taste of gluten-free products, frequently describing them as dry, crumbly, and bland. In this regard, do Nascimento et al. [10] highlight the concept of normality for celiac patients, since finding a product with optimal sensory characteristics means regaining the ability to return to their routines—not just eating, but also avoiding social isolation and feeling normal when sharing a meal.

Conversely, when the stimulus was presented with the “gluten-free” Anglicism, the Health aspects category ranked first, indicating that celiac consumers are susceptible to foreign terminology. This is particularly interesting, since among non-celiac consumers, with both the “*libre de gluten*” and “gluten-free” stimuli, the Health aspects category also had the highest relative frequency, thus suggesting a strong and stable association between the food concept and the idea of health, regardless of the language used. Therefore, Anglicism could evoke aspects related to health and lifestyle, which would cognitively distance the consumer from a specific consumption experience, bringing them closer to a more symbolic and aspirational representation.

However, since the presence of the Aspirational category among celiac consumers for the English term is mentioned with low frequency, this also suggests that its relevance lies in the symbolic value it holds for these consumers. Consequently, this suggests that, in this study, loanwords are not only adopted as medical synonyms but also incorporate sociocultural connotations associated with status, modernity, and lifestyle trends [11,15]. Finally, the Nutritional aspects category received the lowest scores across both languages and groups, in contrast to the high frequency of Health aspects. This behavior provides evidence supporting the effect described by Prada et al. [4], according to which consumers assume that a product is healthy simply because it is gluten-free, ignoring its actual nutritional composition (vitamins, minerals, or fiber). This contradiction is concerning, as gluten-free diets often lack micronutrients and contain higher levels of sugars and fats [6]. Meanwhile, categories such as Market availability, Hedonic aspects, Manufacturing processes, and Legislation showed minimal recall rates in all cases, suggesting a low spontaneous prominence of technological complexity, pleasure, or formal regulation in the participants’ minds.

#### 4.2.2. Structural Representation

An analysis of the internal structure reveals the hierarchical organization of these categories and highlights differences in the cognitive drivers of each group studied. According to the results for celiac consumers in both languages, this group formed a dense and complex core based on terms related to safety and safety assurances, such as Healthy, Safe, and Suitable. According to Abric [33], these elements constitute the most solid foundation of the representation, characterized by their rigidity and resistance to change. A critical finding within this core was the emergence of the term Expensive, which had a marked negative valence. The inclusion of this concept in the central core demonstrates that the economic factor is not a peripheral or transitory aspect for the celiac consumer, but rather a structural element of exclusion that defines their daily dietary identity [10]. This cognitive cost centrality is directly linked to the reality of the Mexican market, where specialized products are significantly more expensive and have limited commercial distribution [6,8].

For non-celiac consumers, the core was structured around a single element: the word Healthy. This confirms the presence of a health halo among non-celiac consumers [1,4,5], revealing a simplified perception that reduces the identity of gluten-free foods to the belief that they are inherently beneficial to health. Regarding the first periphery (Zone 2), which stabilizes the core and acts as an adaptive filter against environmental changes [32,43], an almost total absence of elements was observed. Only celiac consumers incorporated the terms Wheat and Tasteless into this zone with a negative valence.

Following Abric’s theory [43], the lack of terms in the first periphery reveals a rigid and defensive social representation; the fact that wheat does not emerge as a high-frequency element suggests that the celiac community perceives the allergen as a constant threat requiring constant vigilance and a cognitive protection mechanism. For non-celiac consumers, this area remained empty. According to Gómez-Corona et al. [24], a structure lacking peripheral protective elements renders the social representation highly vulnerable to rapid changes in response to informational campaigns that could debunk the current health halo.

In contrast, in the second periphery zones (Zones 3 and 4) it became evident how the use of loanwords functions as a cognitive modulator [16]. In non-celiac individuals, the English stimulus in these zones activated words linked to marketing and lifestyles (Trend, Anti-inflammatory, Light), while the Spanish term mobilized concepts closer to clinical nutritional descriptions (Nutritious, Allergy). For the celiac group, language also divided the periphery: English was linked to institutional and commercial labeling dimensions, promoting the search for elements of external validation, while Spanish anchored the representation in the difficulties of the local, everyday experience. This explains the placement of transitional terms on the border of Zone 4, such as Doubt, Edible, and Tasteless.

The presence of doubt on the periphery reflects an unstable and alert perception, consistent with the reports by Calderón de la Barca et al. [8] on the prevalence of cross-contamination and the lack of official seals on domestic products in Mexico. Finally, Zone 4 contained the negative sensory attributes common to all the studied subgroups, notably Tasteless and Flavorless, along with the base ingredients (Bread, Flour, Wheat). As this is the zone most sensitive to change and dependent on the empirical history of the subjects [30,34], the convergence of these evocations confirms that the technological and sensory limitations of alternative flours constitute the universal empirical barrier for this kind of food, regardless of whether or not consumers are celiacs [9,20].

#### 4.2.3. Correspondence Analysis

Correspondence analysis integrates the previous findings by geometrically projecting the associations and spatial proximity between consumer profiles and verbal stimuli. The two-dimensional map reveals a clear polarization in the factor space, graphically confirming the divergent nature of the social representations studied. Non-celiac consumers are clustered in the quadrant of wellness trends and symbolic market values. Spatially, this profile intersects with terms such as Trend, Light, and Anti-inflammatory. This graphical proximity corroborates the structural fragility identified in their cognitive map; since their spatial position is not linked to variables of biological vulnerability or illness experience, the profile of non-celiacs is represented as a volatile consumer identity, subject to trends in international marketing described by Prada et al. [4].

In contrast, consumers with celiac disease are positioned on the opposite side of the two-dimensional space, consistently drawn towards the quadrant of risk management and medical safety. The cluster analysis of the CA coordinates revealed a close association between the celiac consumer and terms related to biological protection such as Safe and Suitable. However, this graphical search for refuge is strained on the map by the constant proximity of the Expensive vector. The spatial projection demonstrates that rising costs do not behave as an isolated variable, but rather as a transversal axis that intersects the celiac profile in both languages, geometrically visualizing the economic frustration and exclusion documented by Arias-Gastelum et al. [6].

In this vein, for consumers with celiac disease, nutritional and descriptive categories carried considerably less weight, having been supplanted by Consumption experience and Economic aspects. This difference shows that non-celiac consumers construct their perception of the product from a more descriptive and intellectual perspective, focusing on the raw materials and the formulation of the food, while celiac consumers prioritize addressing immediate needs related to safety, accessibility, and sensory acceptability, which aligns with the findings reported by Alencar et al. [3]. Consequently, the exact composition and nutritional quality of the food lose importance, as the daily urgency of adhering to a celiac diet overshadows other aspects, such as the product’s nutritional quality.

The correspondence map clarifies the role of language as a two-dimensional attractor. The Anglicism “gluten-free” may exert a spatial pull that shifts the profiles of celiac consumers towards concepts of institutional validation and commercial prestige, such as Reliable and Certify. This graphic distancing from international reality confirms the thesis of Alcántara-Pilar et al. [16] regarding the transfer of internationally recognized stereotypes through the use of English language. However, the simultaneous appearance of the term Misleading in the vicinity of this same quadrant visually illustrates the celiac consumers’ latent suspicion regarding the promises on the packaging. This behavior would indicate cognitive polyphasia [35,44], a phenomenon which occurs when a single social group harbors contradictory logics, as reflected in the complexity of the reality of the Mexican market for gluten-free products. In this vein, recent studies have shown an inappropriate use of precautionary labeling in the country, demonstrating that up to 17.4% of the commercial products analyzed and labeled as suitable contain more than 20 mg/kg of gluten, including products that bear certifications [8].

In contrast, the Spanish phrase “*libre de gluten*” directs consumers towards the uncertainty quadrant of the local market, becoming spatially linked to the term Doubt. This geometric configuration is a direct reflection of the Mexican health context discussed earlier; the local language connects the consumer to the unregulated experience of their immediate environment, possibly associating it with mistrust regarding cross-contamination control.

Finally, the placement of the unfavorable sensory variables (Tasteless and Flavorless) at the center of the plane or in intersection zones shared by all profiles demonstrate that organoleptic barriers act as a substrate of universal dissatisfaction. Considering the correspondence analysis maps, the phenomenon in Mexico behaved as a divided space: for the non-celiac consumer, the map represents an esthetic, aspirational, and transitory adoption; for the celiac consumer, it represents a constant spatial negotiation between the need for safety, distrust of labeling, and economic penalty.

## 5. Conclusions

This study examined the social representation of the concepts “gluten-free” and “*libre de gluten*” among Mexican populations with and without celiac disease by employing *K*-proportions analysis, a structural approach, and correspondence analysis. The results show that gluten-free foods are interpreted by consumers based on the experiences, needs, and everyday context of each group. While for non-celiac consumers the concept is primarily linked to health and lifestyle, for celiac consumers it relates to accessibility, safety, sensory dissatisfaction, quality of life, and trust. Furthermore, the findings confirm that language functions not only as a translation resource but also as an element that can alter the social representation of food.

The use of the “gluten-free” Anglicism partly triggers symbolic associations linked to status, credibility, and well-being, partially shifting attention away from the concrete experience of consumption. Considering the above, these findings show that in the Mexican context, the consumption of gluten-free products goes beyond nutritional considerations and is influenced by cultural, symbolic, and commercial factors, as well as by trust in product labeling and safety assurances.

Therefore, the consumption of these products is a complex activity involving health, economic, and linguistic factors. In this vein, the present study contributes to exploratory research on the influence of language on the formation of social representations of concepts. Finally, the research highlights a gap between voluntary lifestyle choices and health risks: while for non-celiacs the choice of these products reflects a voluntary pursuit of a certain lifestyle and status, for celiacs it represents a constant balancing act between health risks, dietary restrictions, and economic exclusion.

## 6. Limitations and Future Directions

While the results obtained provide valuable insights into how Mexican consumers interpret and understand the gluten-free concept, the associations identified pertain to a specific sociocultural context and may change based on consumer trends, product availability, and market communication strategies. To build upon these findings, future research could explore how other elements of the commercial environment, such as packaging design, certification seals, or nutritional claims, influence perceptions of safety and trust in gluten-free products among both celiac and non-celiac consumers.

It is also important to note that this was an exploratory study and that people diagnosed with celiac disease, both in Mexico and around the world, represent a small percentage of the population, thus making them underrepresented populations that are difficult to reach. Furthermore, due to the nature of the sampling, it is important to note that it was not possible to obtain a balanced consumer group in terms of sociodemographic variables. Therefore, it is recommended that future research employs balanced sampling with these types of populations. Since the study did not measure English proficiency, it would be advisable to include this variable in future studies, as it could influence the structure of the representation of the term under study. Additionally, from a practical standpoint, the food industry needs to improve the sensory attributes related to the taste and texture of these foods so that celiac patients can regain a sense of normalcy in their social lives. Concurrently, marketing strategies must avoid generating inappropriate health claims that could mislead the general public.

## Figures and Tables

**Table 1:** Sociodemographic characteristics of the surveyed participants (expressed as a percentage).

Characteristics	Condition	
Gender	Celiac	Non-celiac
Female	81.7	63.3
Male	18.3	36.7
Education		
No university	12.5	37.5
University	87.5	62.5
Age		
18–24	10.8	35.0
25–34	22.5	28.3
35–44	29.2	18.3
45–54	20.8	12.5
55 or more	16.7	5.8
Product experience		
Less than 1 year	9.2	23.9
1–3 years	35.0	30.6
4–7 years	28.3	23.1
8–10 years	13.3	5.8
Over 10 years	11.7	16.5
General product familiarity *	5.7 a	4.2 b

* Values with different letters in the same row indicate statistically significant differences (*p* < 0.05).

## Data Availability

The data are available upon justified request to the corresponding author. They are not publicly available due to privacy restrictions.
